# Reducing Prescribing Errors in Paediatric Patients by Assessment and Feedback Targeted at Prescribers

**DOI:** 10.5402/2011/545681

**Published:** 2011-09-20

**Authors:** Michael Eisenhut, Blanche Sun, Sarah Skinner

**Affiliations:** Luton & Dunstable Hospital National Health Service Foundation Trust, Lewsey Road, Luton LU40DZ, UK

## Abstract

Prescribing errors are the most common type of medical errors and can result in harm particularly in young children. Doctors were enrolled in a programme of written assessment in prescribing skills and individualized feedback. Pharmacists audited the impact. The setting was the paediatric wards and neonatal unit of a District General Hospital. 16 doctors were tested and received feedback. A total of 110 errors were identified in this test, out of a 51 were classified as major including wrong dose and frequency, and prescribing medication the patient had an allergy to. Audit of impact of this intervention revealed a reduction of errors from 47 to 21, and patients affected from 19 to 11 per 100 (*P* = 0.001) emergency admissions compared to an audit before the intervention. An intervention combining a comprehensive multifaceted assessment and detailed feedback can lead to reduction of prescribing errors in paediatric trainees.

## 1. Introduction

Medication errors are considered to be the most common type of medical error, and have been defined by the UK Department of Health and US National Coordinating Council for Medication Error and Prevention as “…any preventable event that may cause or lead to inappropriate medication use or patient harm, while the medication is in the control of the health care professional, patient, or consumer.” [1]. The most common form of medication error has, for paediatric patients, been identified as physicians prescribing error [2, 3]. It has been estimated that the incidence of paediatric dosing errors is about 500 000 per year in England [4]. Strategies to reduce errors have included increased input from clinical pharmacists, system change implemented by critical incident analysis, recently computerized physician order entry (CPOE), and computer-aided prescribing [3, 5]. A recent review noted that there were two published examples of assessment of the impact of an educational intervention on prescribing errors in paediatric patients, but it was not clear which part of several interventions reduced errors [6]. The authors of this review demanded more research to establish whether education does indeed reduce prescribing errors. 

Objectives of our project were

1. to reduce prescribing errors putting paediatric patients at risk of harm;
2. to reduce the risk associated with lack of necessary information for safe transcribing;
3. to reduce the risk associated with lack of acting on prescriptions with errors.

## 2. Materials and Methods

### 2.1. Audit Work

A baseline audit was undertaken on all prescribing errors noted on daily (week days) review of all drug charts by pharmacists on their routine visits of the paediatric wards and the neonatal unit for about a month in April and May 2008. All drug charts with incorrect prescribing including nonadherence to guidance given in the British National Formulary for children [7] were photocopied for further analysis and errors documented. Major errors were for the analysis defined as prescribing a drug the patient is allergic to, prescription of the wrong dose or unit, guessing the dose a patient has been on, not prescribing a required drug and choice of the wrong frequency for application of a drug.

### 2.2. Assessment and Feedback for Trainees

Prescribing skills were subsequently assessed by asking paediatric trainee doctors to complete 5 tasks relating to prescribing as part of the induction programme on commencement of their post at Luton and Dunstable Hospital NHS Foundation Trust, United Kingdom. The task comprised of transcribing a drug chart containing deliberate errors, two scenarios requiring prescription, and two tasks with instructions for prescription of intravenous drugs (see Appendix A). Trainees were given one hour to complete the tasks and had a calculator and the British National Formulary for Children with information on all medication and doses including indications, contra-indications, and allergy information available for reference. One pharmacist (B. Sun) and one paediatrician (M. Eisenhut) reviewed the outcome of the assessment with the 5-point questionnaire, and the pharmacist e-mailed assessed trainees with a detailed personalized feedback on correct and incorrect answers for each question individually. All trainees with potentially fatal errors relating to allergy or potentially life-threatening major errors (≥10-fold overdoses) were asked to have all their prescribing checked by a senior doctor until a reassessment with five different tasks (see Appendix B) was completed satisfactorily.

### 2.3. Reaudit and Data Analysis

Two months after this assessment and feedback, a further in-depth audit of prescribing errors was performed in November 2008 using the same approach as the baseline audit. 

Statistical comparison between prescribing errors of audit and re-audit was by reference to the total number of emergency admissions and chi-square test (with Yates correction if sample size was <30) or Fisher's exact test (if sample size was less than 5) with the assumption that major errors in the majority of cases did not occur more than once per patient and with regards to comparison of dose errors; when results of test and retest were compared, more than once per task. Median error rates were compared using the Mann-Whitney test. A *P* value of <0.05 was regarded as indicating statistical significance. Software packages SSPS version 15.0 and Epi Info version 6.04 (Center for Disease Control, Atlanta) were used for statistical analysis.

## 3. Results

### 3.1. Analysis of the Results of Training and Assessment Modules

A total of 16 junior and middle grade doctors underwent assessment and feedback. These constituted the majority (about 80%) of prescribing doctors on paediatric and neonatal units, who change 3 to 6 monthly on training rotations.

Compared to middle grade doctors (more than 2 years experience in pediatrics), junior doctors had overall more errors in prescribing but the same amount of errors concerning prescribing medication the patient was allergic to (see Table 1).

For potentially fatal transcribing errors, it was noted that despite transferring the penicillin allergy information onto the new drug chart 11/16 candidates prescribed co-amoxiclav by reproduction from the template. Doctors who did not commit this type of prescribing error had significantly less other errors than doctors who did (median (range) of 3 (0–5) versus 6 (4–9), *P* = 0.007).

### 3.2. Impact of the Training and Assessment Module on Prescribing Errors

Investigation into the effectiveness of feedback on prescribing errors regarding intravenous drugs used on NICU revealed that, in the two test items requiring calculation of doses for intravenous infusions, in 11 trainees who required retesting, the error rate did not decrease significantly from 10/22 to 8/22 tasks completed *P* = 0.75. None of the trainees had a 10-fold dosing error. All trainees passed the retest.

There was an overall reduction of prescribing errors and number of patients affected but no significant reduction of major drug errors (see Table 2).

In the general paediatric patients, comparison of major drug errors before (28, 7% of emergency admissions) and after (24, 4% of emergency admissions) the intervention revealed a nonsignificant (*P* = 0.08) reduction once corrected for emergency admissions.

In the category of major errors, there was no significant reduction on the neonatal intensive care unit, where there were 11 versus 8 before the intervention indicating a nonsignificant increase from 30 to 41 per hundred emergency admissions to NICU. Other error categories showed significant improvement and included inappropriately prescribing the dose as volume, which occurred in 7 charts of the first and 3 of the second audit. Omissions of dose, unit, frequency, time of administrations, start date, and allergy information occurred in 108 in April/May (26 per 100 emergency admissions) and in 52 in November (9 per 100 emergency admissions), a marked reduction not assessable by statistical analysis because of variable number of errors per patient. The allergy section on the drug chart was not completed in 8 patients of the baseline and 1 patient in the re-audit, which was a significant reduction related to number of emergency admissions (*P* = 0.005, Fisher's exact test). Other infringements in safe prescribing practice including unclear and illegible prescribing were not different between the audits.

## 4. Discussion

Both objectives of a reduction of the number of errors and improved prescribing practice by improvement of information given in the prescription were achieved. We demonstrated an improvement of prescribing practice by less omissions and more attention to information on allergies by introduction of a module of assessment and feedback by a pharmacist. Our rate of 47 errors per 100 emergency admissions noted at the baseline audit was similar to the rate of 55 per hundred admissions reported previously [2]. Our audit could not exclude that the reduction of errors we observed was due to better prescribing skills of a new intake of doctors as the baseline audit was undertaken before their induction. For logistical reasons related to the duty rota, it was only possible to complete assessment and reassessment with feedback during the induction phase. Our audit did only show a nonsignificant reduction of major errors affecting dose. Difficulties in improving calculation skills relating to dilution of drugs and intravenous infusions were also evident from the comparison of results of assessment and reassessment. This mirrored results of projects of other groups published previously noting that there were more dose errors relating to intravenously applied drugs and more drug-related errors on the Neonatal Intensive Care Unit, where most drugs are given intravenously [2, 5]. The root cause for the persistence of this problem may be a deficit in training in relevant calculation skills. Junior doctors perceived this training deficit as significant and deficits in calculation of drug doses have been noted both in US paediatric residents and UK junior doctors [6]. The result of this project, which showed that junior doctors had more than twice as many major prescribing errors compared to more experienced middle grade doctors, demonstrated that these skills can be acquired. Previous studies showed inexperience to be a significant risk factor for medication errors [8]. Improvement in this area may require prolonged training during medical school and integration of assessment of calculation of drug doses in postgraduate medical examinations. Computerized physician order entry (CPOE) has been advocated as a solution to prescribing errors, but it does not apply to emergency and particular resuscitation situations. One study showed that patient mortality rate increased after the introduction of CPOE, and this may have been related to delays in medication administration as more time was needed to enter orders with staff required to spend more time at a computer terminal and less time at the bedside reducing staff-to-patient ratios during critical periods [9]. 

A major concern is that the majority of junior and middle grade doctors transcribed a potentially fatal error of the prescription of co-amoxiclav in a patient they recognized (by putting penicillin in the allergy section) as allergic to penicillin. Not committing this transcription error was associated with a marked reduction in other major errors making this error a marker for decreased ability to reflect on the prescribing process. The significant risk associated with the tendency to reproduce errors during transcribing has been recognized previously, and an assessment which included tasks of transcribing drugs, which were dosed to high, revealed that 60.3% of inappropriately high doses were missed and the error reproduced with only 4/34 participants able to detect all inappropriate doses [10]. Another group found that none of 21 paediatric residents undergoing an assessment were able to identify any of three infusion-related errors in a test [11]. 

Future research needs to determine whether prolonged training in calculation of drug doses and critical transcribing can avoid prescribing errors. This should be integrated in training at the undergraduate and postgraduate level and form part of examinations to provide incentive for enhancement of specific skills in this area in doctors.

## 5. Conclusion

An intervention combining a comprehensive multifaceted assessment and detailed pharmacist-led feedback can lead to a reduction of prescribing errors in paediatric patients. A single intervention to improve prescribing is not sufficient to address calculation errors specific to preparation of intravenously administered drugs.

## Figures and Tables

**Table 1:** Result of assessment of prescribing of paediatric doctors.

	Middle grade (*n* = 8)	Junior grade (*n* = 8)
Total number of errors^1^	45	65
Major errors^2^	10	27
Potentially fatal errors relating to allergy	7	7

^1^Errors affecting dose and frequency, transcribing errors regarding duration, wrong application, missing dose, unit, timing or signature, illegible writing, wrong spelling.

^2^Defined as prescribing a drug the patient is allergic to, prescription of the wrong dose or unit, guessing the dose a patient has been on, not prescribing a required drug, and choice of the wrong frequency for application of a drug.

**Table 2:** Results of audits of all drug charts with errors in prescribing before and after assessment of prescribing skills with feedback in September 2008.

	Audit 21.04.2008 to 16.05.2008 (number of emergency admissions *n* = 421)	Reaudit 01.11.2008 to 30.11.2008 (number of emergency admissions *n* = 588)
Total number of errors (per 100 emergency admissions)	188 (47)	120 (21)^1^
Number of patients with drug errors on their charts (per 100 emergency admissions)	79 (19)	67 (11)^2^
Major errors^3^ (per 100 emergency admissions)	36 (8)	35(6)^4^

^1^Statistical comparison not possible as variable number of errors per admission.

^2^Chi-square test: Chi-square 10.77, *P* value = 0.001.

^3^Defined as prescribing a drug the patient is allergic to, prescription of the wrong dose or unit, guessing the dose a patient has been on, not prescribing a required drug, and choice of the wrong frequency for application of a drug.

^4^Chi-square test: chi-square 2.53, *P* value = 0.11.

## Appendices

### A. Training and Assessment Tool

Instructions: Please complete drug charts with the tasks set and with the help of a BNF for children. If a task cannot be completed with the information given please *state the reason* in the lines below each task.

(1) A drug chart is given (attached) and the information needs to be transferred to a new drug chart.
————————————————————————————————————
————————————————————————————————————
————————————————————————————————————
(2) A 6-year-old child (weight 23 kg) is admitted with respiratory distress and generalized expiratory wheeze and chest pain not responding to paracetamol. Past history reveals that the child is asthmatic and responded previously with bronchospasm to application of nonsteroidal anti-inflammatory drugs. Please fill in a prescription chart with medication addressing the problems (prescribe steroid, bronchodilator as nebulizer, and pain relief).
————————————————————————————————————
————————————————————————————————————
————————————————————————————————————

(3) A 10-year-old child (weight 25 kg) is admitted with inability to swallow because of a peritonsillar abscess. Past medical history reveals that the child is HIV positive, on antiretroviral treatment and has a history of anaphylactic shock in response to ingestion of co-amoxiclav. Please issue a prescription chart with medication addressing the problems.
————————————————————————————————————
————————————————————————————————————
————————————————————————————————————
*For tasks (4) and (5), please specify for each drug the dose range you want to prescribe* (e.g., 10–20 micrograms/kg/hour).
(4) Please prescribe a morphine infusion for a 25-week gestation baby weighing 600 g on the drug chart provided. Dose to be administered is 10 to 20 microgrammes per kg per hour. The nursing staff will use a syringe pump and the drug should be given in a 25 mL volume and 10 microgrammes per kg per hour should be given as 0.1 mL/hour.
————————————————————————————————————
————————————————————————————————————
————————————————————————————————————
(5) Please write a fluid prescription chart for a 1.5 kg, 2-day-old baby, who is on 120 mL/kg/day total daily fluids. The baby is on a morphine infusion running at 0.2 mL/hour and a dopamine infusion at 0.2 mL/hour. The baby needs 10% dextrose and 3 mmol/kg/day sodium and 2 mmol/kg/day potassium. The electrolytes are normal.
————————————————————————————————————
————————————————————————————————————
————————————————————————————————————

### B. Training and Assessment Tool (Retest)

Instructions: Please complete drug charts with the tasks set and with the help of a BNF for children. If a task cannot be completed with the information given, please *state the reason* in the lines below each task.

(1) A parent gives you the following information about his 11-year-old 20 kg child's medication:
Baclofen one tablet three times daily, Tegretol 2 tablets three times a day.
————————————————————————————————————
————————————————————————————————————
————————————————————————————————————
(2) Information given by parents contains only drug names and not doses. What would you do to arrive at an accurate prescription (Give three examples)?
————————————————————————————————————
————————————————————————————————————
————————————————————————————————————
*For tasks (3) and (4), please specify for each drug the dose range you want to prescribe* (e.g., 10–20 micrograms/kg/hour).
(3) Please prescribe a prostaglandin E2 infusion (dinoprostone) to maintain a duct patency in a 2-kg baby with congenital heart disease. Dose is 10 nanogrammes per kg per minute. Dilute in 25 mL normal saline and give at a rate of 2 mL per hour.
————————————————————————————————————
————————————————————————————————————
————————————————————————————————————
(4) Baby H is a 2-day-old 28-week gestation baby. He weighs 800 g. He is hypotensive and needs inotropes. Please prescribe a dopamine infusion dose 10 microgrammes per kg per minute at a rate of 0.2 mL/hour in a total volume of 25 mL of 5% dextrose.
————————————————————————————————————
————————————————————————————————————
————————————————————————————————————
(5) A parent gives you his/her 6-year-old child's medication doses and you are supposed to prescribe the medication on a drug chart as the child has become an inpatient: iron supplement: 5 mL syrup once a day, paracetamol oral suspension 15 mL four times a day, piriton 50 mL four times a day.
————————————————————————————————————
————————————————————————————————————
————————————————————————————————————
